# Do changes in primary care service use over time differ by neighbourhood income? Population-based longitudinal study in British Columbia, Canada

**DOI:** 10.1186/s12939-022-01679-4

**Published:** 2022-06-07

**Authors:** M.R. Lavergne, A. Bodner, S. Peterson, M. Wiedmeyer, D. Rudoler, S. Spencer, E.G. Marshall

**Affiliations:** 1 Department of Family Medicine, Primary Care Research Unit, Dalhousie University, 1465 Brenton Street, Suite 402, Halifax, NS B3J 3T4 Canada; 2 Faculty of Health Sciences, Simon Fraser University, Burnaby, BC Canada; 3 Centre for Health Services and Policy Research, University of British Columbia, Vancouver, BC Canada; 4 Department of Family Practice, University of British Columbia, Vancouver, BC Canada; 5 Centre for Gender and Sexual Health Equity, Vancouver, BC Canada; 6 Faculty of Health Sciences, Ontario Tech University, ON Oshawa, Canada; 7 Ontario Shores Centre for Mental Health Sciences, Whitby, ON Canada; 8 Institute of Health Policy Management and Evaluation, University of Toronto, Toronto, ON Canada

**Keywords:** Health equity, Primary health care, Health services accessibility, Income

## Abstract

**Background:**

Strong primary care systems have been associated with improved health equity. Primary care system reforms in Canada may have had equity implications, but these have not been evaluated. We sought to determine if changes in primary care service use between 1999/2000 and 2017/2018 differ by neighbourhood income in British Columbia.

**Methods:**

We used linked administrative databases to track annual primary care visits, continuity of care, emergency department (ED) visits, specialist referrals, and prescriptions dispensed over time. We use generalized estimating equations to examine differences in the magnitude of change by neighbourhood income quintile, adjusting for age, sex/gender, and comorbidity, and stratified by urban/rural location of residence. We also compared the characteristics of physicians providing care to people living in low- and high-income neighbourhoods at two points in time.

**Results:**

Between 1999/2000 and 2017/8 the average number of primary care visits per person, specialist referrals, and continuity of care fell in both urban and rural settings, while ED visits and prescriptions dispensed increased. Over this period in urban settings, primary care visits, continuity, and specialist referrals fell more rapidly in low vs. high income neighbourhoods (relative change in primary care visits: Incidence Rate Ratio (IRR) 0.881, 95% CI: 0.872, 0.890; continuity: partial regression coefficient -0.92, 95% CI: -1.18, -0.66; specialist referrals: IRR 0.711, 95%CI: 0.696, 0.726), while ED visits increased more rapidly (IRR 1.06, 95% CI: 1.03, 1.09). The percentage of physicians who provide the majority of visits to patients in neighbourhoods in the lower two income quintiles declined from 30.6% to 26.3%.

**Conclusion:**

Results raise concerns that equity in access to primary care has deteriorated in BC. Reforms to primary care that fail to attend to the multidimensional needs of low-income communities may entrench existing inequities. Policies that tailor patterns of funding and allocation of resources in accordance with population needs, and that align accountability measures with equity objectives are needed as part of further reform efforts.

**Supplementary Information:**

The online version contains supplementary material available at 10.1186/s12939-022-01679-4.

## Background

Strong primary care systems are associated with improved health equity [1, 2]. Health equity occurs through the elimination of health disparities, or avoidable differences in health outcomes, between groups positioned with less power in a given social hierarchy [3, 4]. Policies and reforms seeking to address inequities prioritize access and quality of care for people with greater health needs, understanding and attending to the construction of their social position [5, 6]. As morbidity is patterned by income [7, 8], we would expect that in health systems responding to health needs, primary care access would be greater in lower income quintiles [9, 10]. For example, after performance-based remuneration reforms in Turkey, people in the lowest income quintile had 1.30 times the odds of visiting a family doctor versus the highest quintile, even after adjusting for available measures of need [9]. A Canadian study using data from a nationally representative survey found that the frequency of primary care visits, as measured by having more than the median number of primary care visits [10 or more], was higher for respondents with low income [10].

Canadian provinces have undertaken a variety of primary care reforms which may have intended and unintended equity impacts [11], but these have not been explored. Assessing changes in equity requires monitoring the degree and direction that health disparities and their determinants change over time [12]. Equity impacts of primary care reforms have not been studied in this way in Canada, but some evidence suggests people in lower income groups may have benefited less from primary healthcare reforms. In Ontario, enrolment in newly capitated Family Health Networks was lower in the lowest income group [13, 14], as was enrolment in Primary Care Networks among low-income Albertans with diabetes [15]. In Ontario, cancer screening gaps by neighbourhood income quintile grew wider in the context of primary care payment reform [16]. In Quebec, differences in primary care use across income groups persisted but did not change over a period of reform [17]. International evidence has been mixed, with inconsistent findings with respect to the impact of reforms on equity in access in China, Colombia, Brazil, New Zealand, and Sweden [18–21]. In Sweden, where this topic has been thoroughly researched, findings predominantly suggest that the impacts of primary care reforms were more pronounced among groups with relative advantage [21–23]. More broadly, exploration of changes in primary care service use over time has been limited, with a handful of studies exploring physician visits over time [10, 24–26], but few documenting trends across social hierarchies. Studies in Norway and New Zealand have found that inequities in general practitioner service use by income and education decreased over time [20, 27], but whether similar patterns are observed in Canada or other settings is unknown.

Reforms to primary care in British Columbia (BC) over the period from 2001 to 2017 focused on changes to fee codes within the fee-for-service payment system, voluntary patient enrolment programs, practice support for quality improvement, as well as some regional networking of care providers [11, 28]. In the context of fee-for-service payment and voluntary enrolment programs, physicians may be incentivized to provide services to, and enrol, healthier patients with less complex needs, who on average are likely to be people with higher incomes [29, 30]. People in positions of economic advantage also have resources to navigate access to innovative models [31, 32] while people with lower income face documented barriers to primary care access, including access to transportation, availability of care during limited office hours, and having a regular source of care [33–35]. Reforms in BC did not explicitly address barriers to care associated with income [11, 28] and the effect of reforms on use of primary care across income groups has not been studied.

We use linked administrative data to describe trends in primary care services use (number of physician visits, continuity of care, referrals to specialists made in primary care, and emergency department visits) in BC over the period from 1999/2000 to 2017/8. We expect that, as people with lower income experience a higher burden of morbidity, use of primary care services is likely to be socioeconomically patterned, with higher use among people with lower income [9, 10, 24, 29, 30, 36–42]. We determine if the magnitude and direction of any changes in primary care service use differs by neighbourhood income quintile, stratifying analysis between urban and rural/remote settings.

## Methods

### Study setting

The province of British Columbia (BC) was defined on the lands of more than 200 Indigenous nations, through resisted historical and ongoing colonial processes including forced displacement. The legacies of colonialism and racism foundational to the history of Canada tie together income and health outcomes among Indigenous people, Black people, and many racialized people, who are more likely to experience discrimination in economic opportunities as well as higher burdens of morbidity and mortality [43, 44]. Income and health status are further linked as disabled people in BC who rely on government income support programs are compelled to live on an inadequate monthly amount and are prohibited from earning supplemental income or risk losing their minimal supplemental health benefits [45, 46].

The Medical Services Plan (MSP) is BC’s provincial health insurance program that covers health care benefits for eligible BC residents, including people who hold Canadian citizenship and people with permanent residency who meet conditions [47]. Though there are no out of pocket costs at point of care in the Canadian setting for people with provincial insurance, people with lower income face multiple barriers to primary care access [33–35], despite experiencing a higher burden of morbidity and associated need for services.

### Data and study population

We use linked data accessed through Population Data BC and covering all people registered for BC’s provincial health insurance (MSP) at any point over the period from 1999/2000 through to 2017/8 [48–52], including data on all people registered for provincial health insurance (MSP) (a), payments to primary care physicians (b), emergency department visits (c), and prescriptions dispensed (d). Information on hospitalizations was used as part of our measure of comorbidity (e). All inferences, opinions, and conclusions drawn in this manuscript are those of the authors, and do not reflect the opinions or policies of the Data Steward(s).

For analysis of people living in urban settings we selected a random 15% sample of the population, for a total study population of 10,967,280 residents of urban areas and 10,024,616 residents or rural and remote areas. People who are not eligible for provincial insurance, including people with expired or no immigration permits are not included in this analysis. Characteristics of the study samples are described in Additional file 1.

### Measures

#### Primary care service use

##### Ambulatory primary care contacts

We counted primary care contacts as unique combinations of patient, provider, and date, regardless of the number of fee items billed. We excluded contacts for methadone maintenance therapy as these are very frequent for a small number of individuals, and billing guidelines have changed substantially over time. We included contacts in physicians’ offices, home, long-term care, as well as synchronous virtual visits (available in BC since 2014). We excluded contacts that took place in ED or hospital.

##### Continuity of care

We calculated both the Continuity of Care Index (COCI) and the Modified Modified Continuity Index (MMCI). Results were similar and so we report results for the MMCI (0-100 scale), among patients with 3 or more primary care visits.

##### Emergency Department (ED) visits

We identified MSP claims with a service location in the ED or corresponding to fee items billed only in the ED [53], or where a patient was hospitalized with entry via emergency department.

##### Number of prescriptions dispensed

We counted the number of different drugs dispensed per year, at the level of the first 5 digits of the ATC code. We excluded vaccines (J07), vitamins (A11), mineral supplements (A12), tonics (A13) and various (V) categories.

#### Patient characteristics

Age was obtained from BC’s Medical Services Plan (MSP) registration file. Sex/gender is collected at time of MSP registration. The field is labeled “Gender” on the registration form but only the options “M” and “F” are provided. It is not possible to distinguish between assigned sex, legal sex and gender based on this information, so we label this variable “sex/gender.” Neighbourhood income quintile was determined based on census enumeration area of patient residence, assigned using the PCCF+ conversion file [54, 55]. We used the Statistics Canada Statistical Area Classification Metropolitan Influences Zones [56] to group urban settings (census metropolitan areas and agglomerations) and rural/remote settings (areas with strong to no metropolitan influence). We use the Charlson index to measure comorbidities, including ICD 9 and 10-CA codes from both outpatient and inpatient service use [57].

#### Physician characteristics

Physician age, self-identified gender, years since medical degree (MD), and location of MD training (Canadian vs International Medical Graduate (IMG)) were obtained from the College of Physicians and Surgeons of BC [58]. Urban/rural setting is based on the residence of patients seen; physicians are categorized as practicing in an urban setting if the majority of their patient contacts were with patients from urban settings, similarly for rural settings.

### Analysis

We plotted crude service use over all study years across the entire BC population, stratified by income quintile. We also report cross-sectional tabulations of primary care use within the first and last study year by neighbourhood income quintile, age, sex/gender, and Charlson index categories. We stratify analysis by urban and rural settings, given differences in service use across rural/urban settings [31, 32, 39, 40].

We then used generalized estimating equations (GEE) stratified by rural/urban location to examine changes in the rate of service use across all study years, by income quintile. All models account for study year, and an interaction term between income and year to express the total relative change over the full study period. We also controlled for age, sex/gender, an interaction term between age and sex/gender, and Charlson index. Models of ED visits included a dummy variable to capture a change in location coding in 2006/2007 that resulted in a one-time increase in the identification of ED visits. Models exploring changes in continuity used a normal distribution and identity link. All other models used a negative binomial distribution and log link. Analysis was completed using SAS 9.4 GENMOD and findings are reported in accordance with the Strengthening the Reporting of Observational Studies in Epidemiology (STROBE) guidelines. We summarize parameter estimates for the intercept, time (average annual change), income quintile, and interaction between time and income; report rate ratios for primary care visits, ED visits, specialist referrals, and prescriptions; and beta coefficients for linear models of continuity.

Finally, to describe the physician-level context for service delivery for patients in lower income neighbourhoods, we report the number and percentage of physicians who provided a majority of visits to patients in the two lowest income quintiles in the first and last study year, across all primary care physicians, and by physician gender, age, years since MD, location of MD, and urban/rural setting.

## Results

**Fig. 1:**
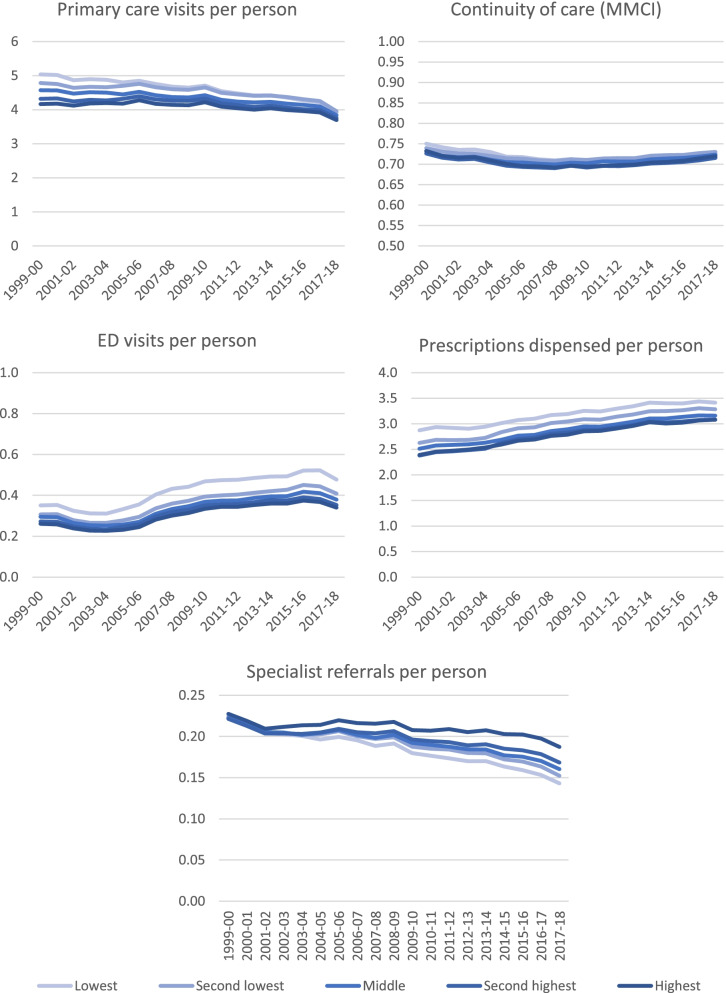
Primary care services use from 1999/2000 to 2017/8, stratified by neighbourhood income quintile

Between 1999/2000 and 2017/8 the average number of primary care visits per person fell from 4.7 to 3.9 in urban settings and from 4.1 to 3.6 in rural/remote settings (Fig. 1, Table 1, Table 2). Continuity fell slightly (from 73.7 to 72.4 in urban settings and from 73.3 to 72.6 in rural/remote settings) and ED visits increased (from 0.29 to 0.36 in urban settings and from 0.35 to 0.60 in rural/remote settings). Specialist referrals fell (from 0.23 to 0.16 in urban settings and from 0.17 to 0.15 in rural/remote settings), while the number of prescriptions dispensed increased (2.6 to 3.2 in urban settings, 2.4 to 3.3 in rural/remote settings) (Fig. 1, Table 1, Table 2).

**Table 1:** Service volume by personal characteristics in 1999/2000 and 2017/8, urban. Mean (SD)

	**Primary care visits per person**		**Continuity of care** **(MMCI – people with 3+ visits)**		**ED visits per person**		**Specialist referrals per person**		**Prescriptions dispensed (ATC 4**^**th**^** level) per person**	
**Year**	1999/2000	2017/8	1999/2000	2017/8	1999/2000	2017/8	1999/2000	2017/8	1999/2000	2017/8
15% BC urban population	4.7 (5.6)	3.9 (4.8)	73.7 (27.1)	72.4 (28.2)	0.29 (0.94)	0.36 (1.12)	0.23 (0.55)	0.16 (0.49)	2.6 (3.4)	3.2 (4.2)
**Neighborhood income quintile**										
1 (lowest)	5.2 (6.2)	4.0 (5.1)	75.2 (26.2)	72.6 (28.2)	0.35 (1.23)	0.45 (1.38)	0.23 (0.56)	0.14 (0.45)	2.9 (3.8)	3.4 (4.6)
2	4.9 (5.7)	4.0 (4.9)	74.0 (26.8)	73.3 (27.8)	0.30 (0.91)	0.37 (1.11)	0.23 (0.55)	0.15 (0.47)	2.6 (3.5)	3.3 (4.3)
3	4.7 (5.6)	3.8 (4.7)	73.3 (27.3)	72.3 (28.2)	0.29 (0.96)	0.36 (1.12)	0.23 (0.54)	0.16 (0.48)	2.5 (3.3)	3.1 (4.2)
4	4.4 (5.1)	3.8 (4.6)	72.5 (27.7)	71.6 (28.4)	0.26 (0.77)	0.32 (0.96)	0.23 (0.54)	0.17 (0.50)	2.4 (3.1)	3.1 (4.0)
5 (highest)	4.2 (5.0)	3.7 (4.6)	73.0 (27.4)	72.0 (28.3)	0.25 (0.75)	0.31 (0.96)	0.24 (0.56)	0.19 (0.54)	2.4 (3.2)	3.1 (3.9)
**Sex/gender**										
F	5.4 (5.9)	4.4 (5.1)	73.9 (26.2)	71.9 (27.8)	0.29 (0.92)	0.37 (1.12)	0.26 (0.59)	0.18 (0.51)	3.0 (3.7)	3.5 (4.3)
M	3.8 (5.1)	3.3 (4.4)	73.3 (28.2)	73.0 (28.7)	0.29 (0.97)	0.36 (1.12)	0.20 (0.51)	0.15 (0.46)	2.1 (3.1)	2.8 (4.0)
**Age (years)**										
0-19	3.2 (3.8)	2.2 (2.9)	60.9 (30.2)	60.3 (31.3)	0.31 (0.77)	0.32 (0.85)	0.12 (0.38)	0.08 (0.30)	1.3 (1.7)	1.1 (1.7)
20-39	4.3 (5.4)	3.0 (4.1)	68.1 (28.1)	61.4 (30.8)	0.28 (0.93)	0.34 (1.16)	0.19 (0.49)	0.09 (0.35)	2.0 (2.6)	2.0 (2.8)
40-59	4.7 (5.7)	3.9 (4.6)	78.1 (24.0)	73.8 (26.8)	0.22 (1.00)	0.31 (1.06)	0.27 (0.59)	0.17 (0.49)	2.8 (3.3)	3.3 (3.9)
60-79	7.2 (6.8)	5.8 (5.6)	86.1 (18.1)	82.0 (21.7)	0.33 (1.02)	0.41 (1.22)	0.42 (0.73)	0.31 (0.68)	5.0 (4.6)	5.8 (5.2)
80+	8.4 (7.5)	8.0 (7.3)	88.3 (16.0)	86.3 (17.8)	0.68 (1.36)	0.83 (1.65)	0.37 (0.69)	0.33 (0.70)	6.6 (5.5)	8.4 (6.2)
**Charlson index**										
0	3.9 (4.7)	2.9 (3.8)	71.7 (28.0)	68.7 (29.7)	0.22 (0.78)	0.26 (0.85)	0.18 (0.48)	0.11 (0.39)	2.0 (2.6)	2.1 (2.8)
1	8.3 (7.0)	6.7 (5.6)	77.5 (24.0)	77.4 (25.1)	0.52 (1.28)	0.53 (1.35)	0.42 (0.72)	0.30 (0.65)	5.2 (4.2)	6.4 (4.7)
2	10.0 (8.4)	7.7 (6.2)	84.4 (18.3)	80.2 (22.5)	0.75 (1.67)	0.69 (1.65)	0.64 (0.87)	0.41 (0.76)	7.0 (5.4)	7.3 (5.6)
3+	11.8 (9.9)	10.0 (7.9)	86.3 (16.3)	84.5 (18.6)	1.44 (2.20)	1.46 (2.63)	0.72 (0.94)	0.52 (0.90)	9.5 (6.6)	11.1 (6.9)

**Table 2:** Service volume by personal characteristics in 1999/2000 and 2017/8, rural. Mean (SD)

	**Primary care visits per person**		**Continuity of care** **(MMCI – people with 3+ visits)**		**ED visits per person**		**Specialist referrals per person**		**Prescriptions dispensed (ATC 4**^**th**^** level) per person**	
**Year**	1999/2000	2017/8	1999/2000	2017/8	1999/2000	2017/8	1999/2000	2017/8	1999/2000	2017/8
Full BC rural population	4.1 (5.4)	3.6 (4.7)	73.3 (26.0)	72.6 (26.7)	0.35 (0.99)	0.60 (1.48)	0.17 (0.46)	0.15 (0.46)	2.4 (3.4)	3.3 (4.3)
**Neighborhood Income quintile**										
1 (lowest)	4.1 (5.5)	3.6 (4.8)	75.1 (25.5)	73.3 (26.8)	0.35 (1.02)	0.67 (1.62)	0.17 (0.47)	0.17 (0.48)	2.5 (3.5)	3.5 (4.6)
2	4.2 (5.5)	3.7 (4.8)	73.9 (25.7)	72.2 (26.8)	0.35 (1.03)	0.62 (1.56)	0.18 (0.48)	0.16 (0.47)	2.5 (3.5)	3.3 (4.4)
3	4.0 (5.3)	3.7 (4.8)	71.5 (26.8)	73.1 (26.5)	0.35 (0.98)	0.58 (1.42)	0.17 (0.46)	0.15 (0.45)	2.4 (3.3)	3.3 (4.3)
4	4.1 (5.4)	3.6 (4.8)	73.1 (25.9)	71.9 (26.7)	0.34 (0.96)	0.58 (1.41)	0.17 (0.46)	0.15 (0.45)	2.4 (3.3)	3.2 (4.2)
5 (highest)	4.0 (5.2)	3.5 (4.7)	72.5 (26.0)	72.4 (26.7)	0.35 (0.97)	0.56 (1.38)	0.14 (0.43)	0.14 (0.44)	2.3 (3.2)	3.1 (4.1)
**Sex/gender**										
F	4.8 (5.8)	4.2 (4.9)	73.0 (25.5)	71.8 (26.5)	0.36 (1.06)	0.62 (1.54)	0.19 (0.49)	0.16 (0.48)	2.9 (3.6)	3.6 (4.5)
M	3.4 (4.9)	3.1 (4.5)	73.6 (26.7)	73.5 (27.0)	0.34 (0.92)	0.58 (1.41)	0.15 (0.43)	0.14 (0.44)	2.0 (3.0)	2.9 (4.1)
**Age (years)**										
0-19	2.6 (3.3)	1.7 (2.5)	61.8 (28.9)	59.9 (30.8)	0.36 (0.80)	0.54 (1.11)	0.08 (0.30)	0.06 (0.26)	1.1 (1.7)	1.0 (1.7)
20-39	3.8 (5.2)	2.7 (3.9)	68.5 (27.1)	63.2 (29.3)	0.37 (1.13)	0.62 (1.51)	0.13 (0.40)	0.08 (0.33)	1.9 (2.7)	2.0 (2.8)
40-59	4.2 (5.6)	3.6 (4.5)	76.3 (24.1)	72.3 (26.4)	0.27 (0.99)	0.53 (1.50)	0.19 (0.49)	0.15 (0.45)	2.6 (3.3)	3.2 (4.0)
60-79	6.4 (6.6)	5.3 (5.4)	82.8 (19.6)	78.5 (22.9)	0.37 (1.00)	0.62 (1.53)	0.32 (0.64)	0.26 (0.60)	4.6 (4.5)	5.4 (5.1)
80+	8.1 (7.7)	7.9 (7.3)	84.6 (17.9)	82.3 (19.5)	0.69 (1.31)	1.17 (2.08)	0.26 (0.57)	0.26 (0.59)	6.1 (5.4)	8.0 (5.9)
**Charlson index**										
0	3.3 (4.4)	2.7 (3.6)	71.4 (27.0)	69.4 (28.3)	0.27 (0.79)	0.46 (1.11)	0.13 (0.40)	0.10 (0.37)	1.8 (2.5)	2.1 (2.9)
1	7.7 (6.9)	6.3 (5.4)	76.4 (23.5)	76.0 (24.3)	0.62 (1.43)	0.90 (1.86)	0.31 (0.62)	0.27 (0.60)	5.2 (4.2)	6.7 (4.7)
2	9.5 (8.2)	7.6 (6.3)	82.1 (19.2)	79.2 (21.6)	0.82 (1.65)	1.08 (2.29)	0.51 (0.77)	0.38 (0.69)	6.6 (5.3)	7.5 (5.6)
3+	12.2 (9.7)	10.2 (7.9)	83.9 (16.5)	82.3 (18.5)	1.61 (2.51)	2.09 (3.21)	0.60 (0.85)	0.47 (0.81)	9.5 (6.5)	11.4 (6.8)

At baseline (1999/2000) in urban settings we observe income gradients with higher service use among people living in lower income neighbourhoods for all measures of service use except specialist referrals (Table 1). In panel models adjusting for age, sex/gender, and comorbidities, rates of service use in urban settings are higher for people living in lower vs the highest income quintiles for all measures (Table 3). At baseline an income gradient is observed in rural/remote settings only for specialist referrals in adjusted models (Table 2, Table 3).

**Table 3:** Results of multivariable panel regression models

	**Primary care visits**	**Continuity of care** **(MMCI - people with 3+ visits)**	**ED visits**	**Specialist referrals**	**Prescriptions dispensed**
	Rate ratio (95% CI)	ß (95% CI)	Rate ratio (95% CI)	Rate ratio (95% CI)	Rate ratio (95% CI)
**Urban**					
Intercept^a^	4.49 (4.47-4.52)	60.0 (59.9-60.2)	0.103 (0.101-0.105)	0.161 (0.159-0.164)	1.97 (1.95-1.98)
Time (average annual change)	0.989 (0.989-0.990)	-0.24 (-0.25- -0.23)	1.014 (1.013-1.015)	0.987 (0.986-0.988)	1.01 (1.01-1.011)
**Neighborhood Income quintile (reference is highest)**					
1 (lowest)	1.11 (1.10-1.12)	1.61 (1.45-1.77)	1.14 (1.12-1.17)	1.08 (1.07-1.09)	1.07 (1.06-1.08)
2	1.08 (1.07-1.09)	1.16 (1.01-1.32)	1.06 (1.05-1.08)	1.07 (1.06-1.08)	1.04 (1.04-1.05)
3	1.06 (1.05-1.07)	0.54 (0.38-0.70)	1.05 (1.04-1.07)	1.06 (1.05-1.08)	1.02 (1.02-1.03)
4	1.03 (1.03-1.04)	-0.01 (-0.17-0.15)	1.02 (1.01-1.03)	1.03 (1.02-1.04)	1.01 (1.00-1.01)
**Relative change in service use from 1999/2000 to 2017/8 (reference is highest income quintile)**					
1	0.881 (0.872-0.890)	-0.92 (-1.18- -0.66)	1.06 (1.03-1.09)	0.711 (0.696-0.726)	0.924 (0.916-0.933)
2	0.924 (0.915-0.933)	0.10 (-0.16-0.36)	1.05 (1.03-1.07)	0.772 (0.756-0.788)	0.955 (0.946-0.963)
3	0.938 (0.929-0.947)	0.38 (0.12-0.64)	1.02 (1.00-1.04)	0.820 (0.803-0.838)	0.972 (0.963-0.981)
4	0.960 (0.951-0.969)	0.57 (0.30-0.83)	1.01 (0.98-1.03)	0.889 (0.871-0.908)	0.992 (0.983-1.001)
**Rural/remote**					
Intercept^a^	4.65 (4.62-4.68)	62.2 (62.0-62.3)	0.173 (0.171-0.176)	0.111 (0.110-0.113)	2.12 (2.11-2.13)
Time (average annual change)	0.988 (0.988-0.989)	-0.22 (-0.23- -0.21)	1.016 (1.016-1.017)	0.992 (0.991-0.992)	1.009 (1.009-1.010)
**Income quintile (reference is highest)**					
1 (lowest)	0.998 (0.992-1.003)	1.19 (1.04-1.34)	0.997 (0.986-1.009)	1.07 (1.06-1.09)	1.009 (1.004-1.014)
2	0.983 (0.978-0.988)	-0.05 (-0.20-0.10)	0.978 (0.967-0.989)	1.11 (1.10-1.13)	0.988 (0.984-0.993)
3	0.991 (0.985-0.996)	-0.69 (-0.84- -0.54)	0.977 (0.967-0.988)	1.10 (1.09-1.11)	0.990 (0.985-0.994)
4	0.987 (0.982-0.992)	-0.47 (-0.62- -0.33)	0.977 (0.966-0.987)	1.04 (1.03-1.05)	0.991 (0.986-0.995)
**Relative change in service use from 1999/2000 to 2017/8 (reference is highest income quintile)**					
1	0.998 (0.989-1.008)	-1.86 (-2.12- -1.60)	1.09 (1.07-1.11)	0.981 (0.960-1.003)	0.991 (0.983-0.999)
2	1.02 (1.01-1.03)	-0.31 (-0.56- -0.05)	1.06 (1.04-1.08)	0.915 (0.896-0.935)	1.02 (1.01-1.03)
3	1.02 (1.01-1.03)	1.20 (0.95-1.45)	1.05 (1.03-1.06)	0.905 (0.886-0.924)	1.01 (1.01-1.02)
4	1.02 (1.01-1.03)	0.13 (-0.12-0.37)	1.04 (1.03-1.06)	0.994 (0.973-1.015)	1.02 (1.01-1.03)

^a^Reflecting healthy female age 20-29. All models controlled for age, sex/gender, an interaction term between age and sex/gender, and Charlson index. Models of ED visits included a dummy variable to capture a change in location coding in 2006/7 that resulted in a one-time increase in the identification of ED visits

Models adjusting for age, sex/gender, and comorbidities (Charlson index) reveal significant differences in relative changes by income quintile between 1999/2000 and 2017/8 for all services in urban settings and for continuity and ED visits in rural settings (Table 3). In urban settings, primary care visits fell by 10% more in the lowest neighborhood income quintile relative to the highest (relative change IRR 0.881, 95%CI: 0.872-0.89). Continuity declined more in the lowest relative to the highest neighborhood income quintile in all settings (relative change lowest vs. highest quintile *urban*: -0.92, 95% CI: -1.18, -0.66, *rural*: -1.86, 95% CI: -2.12, -1.6). ED visits increased in all income quintiles but increased more in lower versus higher neighborhood income quintiles throughout the province (relative change lowest vs highest *urban*: IRR 1.06, 95% CI: 1.03, 1.09, *rural*: 1.09, 95% CI: 1.07, 1.11). Specialist referrals per person have fallen over time in urban settings, but this decline is almost 30% greater in the lowest compared to highest neighborhood income quintile (relative change lowest vs highest: IRR 0.711, 95% CI: 0.696, 0.726). Prescriptions dispensed have increased over time, but this increase was somewhat smaller in the lowest neighborhood income quintile relative to highest in urban settings (relative change lowest vs highest: IRR 0.924, 95%CI 0.916, 0.933).

Over the study period, the percentage of physicians whose practices provide a majority of care to patients in lower income neighbourhoods declined from 30.6 to 26.3 (Table 4). Though a higher percentage of male physicians provide a majority of care to patients in lower income neighbourhoods, the difference by gender narrowed between 1999/2000 and 2017/8. In 2017/8, higher percentages of physicians under age 40 (28.6%), within 10 years of their MD (30.2%), and in rural settings (33.0%) provide a majority of care to patients in lower income neighbourhoods. In 1999/2000 higher percentages of physicians over the age of 60, more than 30 years since their MD, or who were international medical graduates provided a majority of care to patients in lower income neighbourhoods, but that was no longer the case in 2017/8.

**Table 4:** Primary care physicians providing majority care to patients in lower income neighbourhoods (lowest two quintiles)

	**1999/2000** (*n *= 4,122)	**2017/8** (*n *= 6,068)
Overall - N (%)	1,260 (30.6)	1,584 (26.3)
**Gender - N (%)**		
Female	297 (23.6)	631 (24.3)
Male	963 (33.7)	953 (27.8)
**Age - N (%)**		
< 40	480 (31.4)	501 (28.6)
40-49	374 (27.4)	344 (25.4)
50-59	248 (29.5)	398 (24.3)
>= 60	158 (40.6)	341 (26.6)
**Years since MD - N (%)**		
<10	310 (32.2)	398 (30.2)
10 to 19	396 (29.0)	340 (25.1)
20 to 29	311 (27.6)	361 (24.8)
30+	243 (36.3)	485 (25.5)
**Location of MD - N (%)**		
Canada	917 (29.3)	1,044 (26.1)
IMG	321 (35.1)	497 (26.3)
**Urban/rural - N (%)**		
Urban (MIZ 1-3)	160 (31.1)	248 (33.0)
Rural (MIZ 4-7)	1,100 (30.5)	1,336 (25.3)

## Discussion

Between 1999/2000 and 2017/8 the average number of primary care visits per person, specialist referrals, and continuity of care fell in both urban and rural settings primary care, while ED visits and prescriptions dispensed increased. At baseline, we observe higher service use for all measures among people living in lower compared to higher neighborhood income quintiles in urban settings, and only for specialist referrals in rural settings. However, findings reveal substantial differences in relative changes by neighborhood income quintile in urban settings. Primary care visits, continuity, and specialist referrals declined more rapidly for people in the lowest relative to the highest neighborhood income quintiles, while ED visits increased faster. There is now a pronounced income gradient in who receives specialist referrals, favouring residents of wealthier neighbourhoods. Prescriptions dispensed have increased over time, but this increase was somewhat smaller in the lowest neighborhood income quintile relative to highest.

We expected that at baseline, services use would be higher among people living in lower-income neighbourhoods [9, 10, 24, 29, 30, 36–42], as social contexts have a well-recognized impact on health [59]. Legacies of colonialism and racism [43, 44], ableism and inadequate support for people with disabilities [45, 46], and poverty among seniors [60] further tie together income with morbidity and need for health services. While there are no specific guidelines about the appropriate number of visits per patient [61, 62], given the context of people living in lower neighborhood income quintiles, the faster decline in visit frequency and continuity, especially adjusting for comorbidity, requires remedy. Taken as a whole, these results describe a deterioration in access to primary care, particularly affecting people living in low-income neighborhoods over the past two decades in BC and appear consistent with emerging evidence from other provinces [3–6, 11, 12].

The overall trend of declining primary care services over time is a somewhat unexpected observation, particularly given an aging population and greater complexity in community-based service delivery. This is, however, consistent with persistent reports of primary care access challenges [63] and declining family physician visit volume described elsewhere [64]. Taken together, findings point to a need for substantial policy changes in primary care to address both declining access overall, and increasing inequity in access. Lavoie and colleagues studied policy requirements for equity-informed primary healthcare services including 1) use of accountability measures aligned with equity objectives and 2) patterns of funding and allocation of resources that are tailored to population needs [65]. These were proposed in the context of Community Health Centres, but may be relevant to the broader range of reforms. In contrast, the province of BC relied largely on voluntary reforms, where physicians choose whether they want to participate as well as for which patient they choose to accept longitudinal responsibility. The effect has been that reform programs – and the investments that support them – have disproportionately impacted patients who live in higher income neighbourhoods [66]. Analysis also shows, for programs that have eligibility requirements related to health status, disparities in resource distribution were less pronounced [66]. These findings are consistent with our observations of growing inequities across the population, and call for reforms that are responsive to patient need, and inequities in access.

A strength of this research is that there were no changes to insurance coverage and only limited changes to physician payment [67] over the study period in BC. This means we are confident in the consistency of measures of primary care use over time. Our income measure is limited to neighbourhood quintiles. It is now possible to identify individuals with low income using data on prescription drug insurance in BC, but this was not available for the full study period [68]. In the data used for this analysis it is also not possible to measure individual or neighbourhood education, racialization, access to housing, immigration status, or other important facets of social position correlated with income that may also shape access to care. However, this does not threaten the internal validity of our analysis as our objective was to examine changes over time by income quintile, and not to estimate the independent relationship between income and service use. Future research should interrogate the health impacts of converging forces of marginalization (e.g., colonialism, racism, housing, migration), with input from people most impacted by these forces to inform recommended actions [69].

## Conclusion

Results raise concerns that equity in access to primary care has deteriorated in BC and suggest that reforms to primary care that fail to attend to the multidimensional needs of low-income communities may further entrench existing inequities. Future reforms should ensure that patterns of funding and allocation of resources are tailored to population needs, and include equity as part of accountability systems. Observed trends are possibly specific to BC primary care context, though findings highlight the need to repeatedly evaluate services use and policy implementation over time with explicit consideration of the root causes of inequities and necessary actions to remedy them.

## Supplementary Information

**Additional file 1.**

## Data Availability

The data that support the findings of this study are approved for use by data stewards and accessed through a process managed by Population Data BC. The data sets used for this study will be archived, and requests for access to them in the context of verification of study findings can be made to PopData (https://www.popdata.bc.ca/data_access). We are not permitted to share the research extract used in this analysis with other researchers, but the same datasets are accessible via Population Data BC.
