# Supplementary material for: Do changes in primary care service use over time differ by neighbourhood income? Population-based longitudinal study in British Columbia, Canada
Source: Int J Equity Health. 2022 Jun 7;21:80. doi: 10.1186/s12939-022-01679-4 (PMC9175477; doi:10.1186/s12939-022-01679-4)
Supplement: Supplementary file 1 — Additional file 1. [file 12939_2022_1679_MOESM1_ESM.docx]

**Title:** Do changes in primary care service use over time differ by neighbourhood income? Population-based longitudinal study in British Columbia, Canada

Lavergne MR^1^*, Bodner A^2^, Peterson S^3^, Wiedmeyer M^4,5^, Rudoler D^6,7^, Spencer S^2^, Marshall EG^1^

1 Department of Family Medicine, Dalhousie University | Halifax, Nova Scotia

2 Faculty of Health Sciences, Simon Fraser University | Burnaby, British Columbia

3 Centre for Health Services and Policy Research, University of British Columbia | Vancouver, British Columbia

4 Department of Family Practice, University of British Columbia | Vancouver, British Columbia

5 Centre for Gender and Sexual Health Equity | Vancouver, British Columbia

6 Faculty of Health Sciences, University of Ontario Institute of Technology | Oshawa, Ontario

7 Institute of Health Policy, Management and Evaluation, University of Toronto | Toronto, Ontario

* ruth.lavergne@dal.ca

Primary Care Research Unit, Dalhousie University
1465 Brenton Street, Suite 402
Halifax NS, B3J 3T4

**Abstract**

**Background:** Though strong primary care systems have been associated with improved health equity, and primary care system reforms in Canada may have had equity implications, these have not been evaluated. We sought to determine if changes in primary care service use between 1999/2000 and 2017/2018 differ by neighbourhood income in British Columbia.

**Methods** We used linked administrative databases to track annual primary care visits, continuity of care, emergency department (ED) visits, specialist referrals, and prescriptions dispensed over time. We use generalized estimating equations to examine differences in the magnitude of change by neighbourhood income quintile, adjusting for age, sex/gender, and comorbidity, and stratified by urban/rural location of residence. We also compared the characteristics of physicians providing care to people living in low- and high-income neighbourhoods at two points in time.

**Results:** Between 1999/2000 and 2017/8 the average number of primary care visits per person, specialist referrals, and continuity of care fell in both urban and rural settings, while ED visits and prescriptions dispensed increased. Over this period in urban settings, primary care visits, continuity, and specialist referrals fell more rapidly in low vs. high income neighbourhoods (relative change in primary care visits: Incidence Rate Ratio (IRR) 0.881, 95% CI: 0.872, 0.890; continuity: partial regression coefficient -0.92, 95% CI: -1.18, -0.66; specialist referrals: IRR 0.711, 95%CI: 0.696, 0.726), while ED visits increased more rapidly (relative change IRR 1.06, 95% CI: 1.03, 1.09). The percentage of physicians who provide the majority of visits to patients in neighbourhoods in the lower two income quintiles declined from 30.6% to 26.3%.

**Conclusion:** Results raise concerns that equity in access to primary care has deteriorated in BC. Reforms to primary care that fail to attend to the multidimensional needs of low-income communities may entrench existing inequities. Policies that tailor patterns of funding and allocation of resources in accordance with population needs, and that align accountability measures with equity objectives are needed as part of further reform efforts.

**MeSH keywords:** health equity; primary health care; health services accessibility; income

**Background**

Strong primary care systems are associated with improved health equity (1,2). Health equity occurs through the elimination of health disparities, or avoidable differences in health outcomes, between groups positioned with less power in a given social hierarchy (3,4). Policies and reforms seeking to address inequities prioritize access and quality of care for people with greater health needs, understanding and attending to the construction of their social position (5,6). As morbidity is patterned by income (7,8), we would expect that in health systems responding to health needs, primary care access would be greater in lower income quintiles (9,10). For example, after performance-based remuneration reforms in Turkey, people in the lowest income quintile had 1.30 times the odds of visiting a family doctor versus the highest quintile, even after adjusting for available measures of need (9). A Canadian study using data from a nationally representative survey found that the frequency of primary care visits, as measured by having more than the median number of primary care visits (10 or more), was higher for respondents with low income (10).

Canadian provinces have undertaken a variety of primary care reforms which may have intended and unintended equity impacts (11), but these have not been explored. Assessing changes in equity requires monitoring the degree and direction that health disparities and their determinants change over time (12). Equity impacts of primary care reforms have not been studied in this way in Canada, but some evidence suggests people in lower income groups may have benefited less from primary healthcare reforms. In Ontario, enrolment in newly capitated Family Health Networks was lower in the lowest income group (13,14), as was enrolment in Primary Care Networks among low-income Albertans with diabetes (15). In Ontario, cancer screening gaps by neighbourhood income quintile grew wider in the context of primary care payment reform (16). In Quebec, differences in primary care use across income groups persisted but did not change over a period of reform (17). International evidence has been mixed, with inconsistent findings with respect to the impact of reforms on equity in access in China, Colombia, Brazil, New Zealand, and Sweden (18–21). In Sweden, where this topic has been thoroughly researched, findings predominantly suggest that the impacts of primary care reforms were more pronounced among groups with relative advantage (21–23). More broadly, exploration of changes in primary care service use over time has been limited, with a handful of studies exploring physician visits over time (10,24–26), but few documenting trends across social hierarchies. Studies in Norway and New Zealand have found that inequities in general practitioner service use by income and education decreased over time (20,27), but whether similar patterns are observed in Canada or other settings is unknown.

Reforms to primary care in British Columbia (BC) over the period from 2001 to 2017 focused on changes to fee codes within the fee-for-service payment system, voluntary patient enrolment programs, practice support for quality improvement, as well as some regional networking of care providers (11,28). In the context of fee-for-service payment and voluntary enrolment programs, physicians may be incentivized to provide services to, and enrol, healthier patients with less complex needs, who on average are likely to be people with higher incomes (29,30). People in positions of economic advantage also have resources to navigate access to innovative models (31,32) while people with lower income face documented barriers to primary care access, including access to transportation, availability of care during limited office hours, and having a regular source of care (33–35). Reforms in BC did not explicitly address barriers to care associated with income (11,28) and the effect of reforms on use of primary care across income groups has not been studied.

We use linked administrative data to describe trends in primary care services use (number of physician visits, continuity of care, referrals to specialists made in primary care, and emergency department visits) in BC over the period from 1999/2000 to 2017/8. We expect that, as people with lower income experience a higher burden of morbidity, use of primary care services is likely to be socioeconomically patterned, with higher use among people with lower income (9,10,24,29,30,36–42). We determine if the magnitude and direction of any changes in primary care service use differs by neighbourhood income quintile, stratifying analysis between urban and rural/remote settings.

**Methods**

***Study setting***

The province of British Columbia (BC) was defined on the lands of more than 200 Indigenous nations, through resisted historical and ongoing colonial processes including forced displacement. The legacies of colonialism and racism foundational to the history of Canada tie together income and health outcomes among Indigenous people, Black people, and many racialized people, who are more likely to experience discrimination in economic opportunities as well as higher burdens of morbidity and mortality (43,44). Income and health status are further linked as disabled people in BC who rely on government income support programs are compelled to live on an inadequate monthly amount and are prohibited from earning supplemental income or risk losing their minimal supplemental health benefits (45,46).

The Medical Services Plan (MSP) is BC’s provincial health insurance program that covers health care benefits for eligible BC residents, including people who hold Canadian citizenship and people with permanent residency who meet conditions (47). Though there are no out of pocket costs at point of care in the Canadian setting for people with provincial insurance, people with lower income face multiple barriers to primary care access (33–35), despite experiencing a higher burden of morbidity and associated need for services.

***Data and study population***

We use linked data accessed through Population Data BC and covering all people registered for BC’s provincial health insurance (MSP) at any point over the period from 1999/2000 through to 2017/8 (48–52), including data on all people registered for provincial health insurance (MSP) (a), payments to primary care physicians (b), emergency department visits (c), and prescriptions dispensed (d). Information on hospitalizations was used as part of our measure of comorbidity (e). All inferences, opinions, and conclusions drawn in this manuscript are those of the authors, and do not reflect the opinions or policies of the Data Steward(s).

For analysis of people living in urban settings we selected a random 15% sample of the population, for a total study population of 10,967,280 residents of urban areas and 10,024,616 residents or rural and remote areas. People who are not eligible for provincial insurance, including people with expired or no immigration permits are not included in this analysis. Characteristics of the study samples are described in Appendix 1.

***Measures***

*Primary care service use*

*Ambulatory primary care contacts:* We counted primary care contacts as unique combinations of patient, provider, and date, regardless of the number of fee items billed. We excluded contacts for methadone maintenance therapy as these are very frequent for a small number of individuals, and billing guidelines have changed substantially over time. We included contacts in physicians’ offices, home, long-term care, as well as synchronous virtual visits (available in BC since 2014). We excluded contacts that took place in ED or hospital.

*Continuity of care:* We calculated both the Continuity of Care Index (COCI) and the Modified Modified Continuity Index (MMCI). Results were similar and so we report results for the MMCI (0-100 scale), among patients with 3 or more primary care visits.

*Emergency Department (ED) visits:* We identified MSP claims with a service location in the ED or corresponding to fee items billed only in the ED (53), or where a patient was hospitalized with entry via emergency department.

*Number of prescriptions dispensed:* We counted the number of different drugs dispensed per year, at the level of the first 5 digits of the ATC code. We excluded vaccines (J07), vitamins (A11, mineral supplements (A12), tonics (A13) and various (V) categories.

*Patient characteristics*

Age was obtained from BC’s Medical Services Plan (MSP) registration file. Sex/gender is collected at time of MSP registration. The field is labeled “Gender” on the registration form but only the options “M” and “F” are provided. It is not possible to distinguish between assigned sex, legal sex and gender based on this information, so we label this variable “sex/gender.” Neighbourhood income quintile was determined based on census enumeration area of patient residence, assigned using the PCCF+ conversion file (54,55). We used the Statistics Canada Statistical Area Classification Metropolitan Influences Zones (56) to group urban settings (census metropolitan areas and agglomerations) and rural/remote settings (areas with strong to no metropolitan influence). We use the Charlson index to measure comorbidities, including ICD 9 and 10 codes from both outpatient and inpatient service use (57).

*Physician characteristics*

Physician age, self-identified gender, years since medical degree (MD), and location of MD training (Canadian vs International Medical Graduate (IMG)) were obtained from the College of Physicians and Surgeons of BC (58). Urban/rural setting is based on the residence of patients seen; physicians are categorized as practicing in an urban setting if the majority of their patient contacts were with patients from urban settings, similarly for rural settings.

***Analysis***

We plotted crude service use over all study years across the entire BC population, stratified by income quintile. We also report cross-sectional tabulations of primary care use within the first and last study year by neighbourhood income quintile, age, sex/gender, and Charlson index categories. We stratify analysis by urban and rural settings, given differences in service use across rural/urban settings (31,32,39,40).

We then used generalized estimating equations (GEE) stratified by rural/urban location to examine changes in the rate of service use across all study years, by income quintile. All models account for study year, and an interaction term between income and year to express the total relative change over the full study period. We also controlled for age, sex/gender, an interaction term between age and sex/gender, and Charlson index. Models of ED visits included a dummy variable to capture a change in location coding in 2006/2007 that resulted in a one-time increase in the identification of ED visits. Models exploring changes in continuity used a normal distribution and identity link. All other models used a negative binomial distribution and log link. Analysis was completed using SAS 9.4 GENMOD and findings are reported in accordance with the Strengthening the Reporting of Observational Studies in Epidemiology (STROBE) guidelines. We summarize parameter estimates for the intercept, time (average annual change), income quintile, and interaction between time and income; report rate ratios for primary care visits, ED visits, specialist referrals, and prescriptions; and beta coefficients for linear models of continuity.

Finally, to describe the physician-level context for service delivery for patients in lower income neighbourhoods, we report the number and percentage of physicians who provided a majority of visits to patients in the two lowest income quintiles in the first and last study year, across all primary care physicians, and by physician gender, age, years since MD, location of MD, and urban/rural setting.

**Results**

Between 1999/2000 and 2017/8 the average number of primary care visits per person fell from 4.7 to 3.9 in urban settings and from 4.1 to 3.6 in rural/remote settings (Figure 1, Table 1a, Table 1b). Continuity fell slightly (from 73.7 to 72.4 in urban settings and from 73.3 to 72.6 in rural/remote settings) and ED visits increased (from 0.29 to 0.36 in urban settings and from 0.35 to 0.60 in rural/remote settings). Specialist referrals fell (from 0.23 to 0.16 in urban settings and from 0.17 to 0.15 in rural/remote settings), while the number of prescriptions dispensed increased (2.6 to 3.2 in urban settings, 2.4 to 3.3 in rural/remote settings) (Figure 1, Table 1a, Table 1b).

**[Insert figure 1 here]**

**Figure 1.** Primary care services use from 1999/2000 to 2017/8, stratified by neighbourhood income quintile

At baseline (1999/2000) in urban settings we observe income gradients with higher service use among people living in lower income neighbourhoods for all measures of service use except specialist referrals (Table 1a). In panel models adjusting for age, sex/gender, and comorbidities, rates of service use in urban settings are higher for people living in lower vs the highest income quintiles for all measures (Table 2). At baseline an income gradient is observed in rural/remote settings only for specialist referrals in adjusted models (Table 1b, Table 2).

Models adjusting for age, sex/gender, and comorbidities (Charlson index) reveal significant differences in relative changes by income quintile between 1999/2000 and 2017/8 for all services in urban settings and for continuity and ED visits in rural settings (Table 2). In urban settings, primary care visits fell by 10% more in the lowest neighborhood income quintile relative to the highest (relative change IRR 0.881, 95%CI: 0.872-0.89). Continuity declined more in the lowest relative to the highest neighborhood income quintile in all settings (relative change lowest vs. highest quintile *urban*: -0.92, 95% CI: -1.18, -0.66, *rural*: -1.86, 95% CI: -2.12, -1.6). ED visits increased in all income quintiles but increased more in lower versus higher neighborhood income quintiles throughout the province (relative change lowest vs highest *urban*: IRR 1.06, 95% CI: 1.03, 1.09, *rural*: 1.09, 95% CI: 1.07, 1.11). Specialist referrals per person have fallen over time in urban settings, but this decline is almost 30% greater in the lowest compared to highest neighborhood income quintile (relative change lowest vs highest: IRR 0.711, 95% CI: 0.696, 0.726). Prescriptions dispensed have increased over time, but this increase was somewhat smaller in the lowest neighborhood income quintile relative to highest in urban settings (relative change lowest vs highest: IRR 0.924, 95%CI 0.916, 0.933).

Over the study period, the percentage of physicians whose practices provide a majority of care to patients in lower income neighbourhoods declined from 30.6 to 26.3 (Table 3). Though a higher percentage of male physicians provide a majority of care to patients in lower income neighbourhoods, the difference by gender narrowed between 1999/2000 and 2017/8. In 2017/8, higher percentages of physicians under age 40 (28.6%), within 10 years of their MD (30.2%), and in rural settings (33.0%) provide a majority of care to patients in lower income neighbourhoods. In 1999/2000 higher percentages of physicians over the age of 60, more than 30 years since their MD, or who were international medical graduates provided a majority of care to patients in lower income neighbourhoods, but that was no longer the case in 2017/8.

**Table 3**. Primary care physicians providing majority care to patients in lower income neighbourhoods (lowest two quintiles)

|  | **1999/2000**  (n=4,122) | **2017/8**  (n=6,068) |
| --- | --- | --- |
| Overall - % | 1,260 (30.6) | 1,584 (26.3) |
| **Gender - %** |  |  |
| Female | 297 (23.6) | 631 (24.3) |
| Male | 963 (33.7) | 953 (27.8) |
| **Age - N (%)** |  |  |
| < 40 | 480 (31.4) | 501 (28.6) |
| 40-49 | 374 (27.4) | 344 (25.4) |
| 50-59 | 248 (29.5) | 398 (24.3) |
| >= 60 | 158 (40.6) | 341 (26.6) |
| **Years since MD - %** |  |  |
| <10 | 310 (32.2) | 398 (30.2) |
| 10 to 19 | 396 (29.0) | 340 (25.1) |
| 20 to 29 | 311 (27.6) | 361 (24.8) |
| 30+ | 243 (36.3) | 485 (25.5) |
| **Location of MD - %** |  |  |
| Canada | 917 (29.3) | 1,044 (26.1) |
| IMG | 321 (35.1) | 497 (26.3) |
| **Urban/rural - %** |  |  |
| Urban (MIZ 1-3) | 160 (31.1) | 248 (33.0) |
| Rural (MIZ 4-7) | 1,100 (30.5) | 1,336 (25.3) |

**Discussion**

Between 1999/2000 and 2017/8 the average number of primary care visits per person, specialist referrals, and continuity of care fell in both urban and rural settings primary care, while ED visits and prescriptions dispensed increased. At baseline, we observe higher service use for all measures among people living in lower compared to higher neighborhood income quintiles in urban settings, and only for specialist referrals in rural settings. However, findings reveal substantial differences in relative changes by neighborhood income quintile in urban settings. Primary care visits, continuity, and specialist referrals declined more rapidly for people in the lowest relative to the highest neighborhood income quintiles, while ED visits increased faster. There is now a pronounced income gradient in who receives specialist referrals, favouring residents of wealthier neighbourhoods. Prescriptions dispensed have increased over time, but this increase was somewhat smaller in the lowest neighborhood income quintile relative to highest.

We expected that at baseline, services use would be higher among people living in lower-income neighbourhoods (9,10,24,29,30,36–42), as social contexts have a well-recognized impact on health (59). Legacies of colonialism and racism (43,44), ableism and inadequate support for people with disabilities (45,46), and poverty among seniors (60) further tie together income with morbidity and need for health services. While there are no specific guidelines about the appropriate number of visits per patient (61,62), given the context of people living in lower neighborhood income quintiles, the faster decline in visit frequency and continuity, especially adjusting for comorbidity, requires remedy. Taken as a whole, these results describe a deterioration in access to primary care, particularly affecting people living in low-income neighborhoods over the past two decades in BC and appear consistent with emerging evidence from other provinces (3–6,11,12).

The overall trend of declining primary care services over time is a somewhat unexpected observation, particularly given an aging population and greater complexity in community-based service delivery. This is, however, consistent with persistent reports of primary care access challenges (63) and declining family physician visit volume described elsewhere (64). Taken together, findings point to a need for substantial policy changes in primary care to address both declining access overall, and increasing inequity in access. Lavoie and colleagues studied policy requirements for equity-informed primary healthcare services including 1) use of accountability measures aligned with equity objectives and 2) patterns of funding and allocation of resources that are tailored to population needs (65). These were proposed in the context of Community Health Centres, but may be relevant to the broader range of reforms. In contrast, the province of BC relied largely on voluntary reforms, where physicians choose whether they want to participate as well as for which patient they choose to accept longitudinal responsibility. The effect has been that reform programs – and the investments that support them – have disproportionately impacted patients who live in higher income neighbourhoods (66). Analysis also shows, for programs that have eligibility requirements related to health status, disparities in resource distribution were less pronounced (66). These findings are consistent with our observations of growing inequities across the population, and call for reforms that are responsive to patient need, and inequities in access.

A strength of this research is that there were no changes to insurance coverage and only limited changes to physician payment [(68)](https://www.zotero.org/google-docs/?E1Q1oY) over the study period in BC. This means we are confident in the consistency of measures of primary care use over time. Our income measure is limited to neighbourhood quintiles. It is now possible to identify individuals with low income using data on prescription drug insurance in BC, but this was not available for the full study period (67). In the data used for this analysis it is also not possible to measure individual or neighbourhood education, racialization, access to housing, immigration status, or other important facets of social position correlated with income that may also shape access to care. However, this does not threaten the internal validity of our analysis as our objective was to examine changes over time by income quintile, and not to estimate the independent relationship between income and service use. Future research should interrogate the health impacts of converging forces of marginalization (e.g., colonialism, racism, housing, migration), with input from people most impacted by these forces to inform recommended actions (69).

**Conclusion**

Results raise concerns that equity in access to primary care has deteriorated in BC and suggest that reforms to primary care that fail to attend to the multidimensional needs of low-income communities may further entrench existing inequities. Future reforms should ensure that patterns of funding and allocation of resources are tailored to population needs, and include equity as part of accountability systems. Observed trends are possibly specific to BC primary care context, though findings highlight the need to repeatedly evaluate services use and policy implementation over time with explicit consideration of the root causes of inequities and necessary actions to remedy them.

**Declarations**

*Ethics approval and consent to participate*

This study has received ethical approval from the Simon Fraser University Office of Research Ethics with harmonised approval from the University of British Columbia, the University of Ottawa, the University of Western Ontario, the University of Ontario Institute of Technology and the Nova Scotia Health Authority.

*Consent for publication*

Not applicable

*Availability of data and materials*

The data that support the findings of this study are approved for use by data stewards and accessed through a process managed by Population Data BC. The data sets used for this study will be archived, and requests for access to them in the context of verification of study findings can be made to PopData (<https://www.popdata.bc.ca/data_access>). We are not permitted to share the research extract used in this analysis with other researchers, but the same datasets are accessible via Population Data BC.

*Competing Interests*

The authors declare that they have no competing interests.

*Funding*

This study was supported by Canadian Institutes of Health Research (R-PJT-155965).

*Authors’ Contributions*

MR Lavergne conceived of the paper and led drafting of the manuscript. A Bodner prepared the literature review and contributed to drafting the manuscript. S Peterson completed analysis. All authors, including M Wiedmeyer, D Rudoler, S Spencer, and EG Marshall contributed to planning, analysis, critical interpretation of findings, and approved the final manuscript.

*Acknowledgements*

All inferences, opinions, and conclusions drawn in this manuscript are those of the authors, and do not reflect the opinions or policies of the Data Steward(s).

**References**

1. Starfield B, Shi L, Macinko J. Contribution of Primary Care to Health Systems and Health. Milbank Q. 2005;83(3):457–502.

2. Kruk ME, Porignon D, Rockers PC, Van Lerberghe W. The contribution of primary care to health and health systems in low- and middle-income countries: A critical review of major primary care initiatives. Soc Sci Med. 2010 Mar 1;70(6):904–11.

3. Braveman PA. Monitoring Equity in Health and Healthcare: A Conceptual Framework. J Health Popul Nutr. 2003;21(3):181–92.

4. Braveman P. Health disparities and health equity: Concepts and measurement. Annu Rev Public Health. 2006;27(1):167–94.

5. Macinko J, Lima-Costa MF. Horizontal equity in health care utilization in Brazil, 1998-2008. Int J Equity Health. 2012 Jun 21;11:33.

6. Nyamande FN, Mosquera PA, San Sebastián M, Gustafsson PE. Intersectional equity in health care: assessing complex inequities in primary and secondary care utilization by gender and education in northern Sweden. Int J Equity Health. 2020 Sep 11;19(1):159.

7. Vanzella-Yang A, Veenstra G. Family income and health in Canada: a longitudinal study of stability and change. BMC Public Health. 2021 Feb 10;21(1):333.

8. Harvey J, Hynes G, Pichora E. Trends in Income-Related Health Inequalities in Canada. Healthc Q. 2016 Jan 29;18(4):12–4.

9. Sözmen K, Ünal B. Explaining inequalities in Health Care Utilization among Turkish adults: Findings from Health Survey 2008. Health Policy. 2016 Jan 1;120(1):100–10.

10. Glazier RH, Agha MM, Moineddin R, Sibley LM. Universal Health Insurance and Equity in Primary Care and Specialist Office Visits: A Population-Based Study. Ann Fam Med. 2009 Sep 1;7(5):396–405.

11. Hutchison B, Levesque JF, Strumpf E, Coyle N. Primary Health Care in Canada: Systems in Motion. Milbank Q. 2011;89(2):256–88.

12. Penman-Aguilar A, Talih M, Huang D, Moonesinghe R, Bouye K, Beckles G. Measurement of Health Disparities, Health Inequities, and Social Determinants of Health to Support the Advancement of Health Equity. J Public Health Manag Pract JPHMP. 2016;22(Suppl 1):S33–42.

13. Sibley LM, Glazier RH. Evaluation of the equity of age–sex adjusted primary care capitation payments in Ontario, Canada. Health Policy. 2012 Feb 1;104(2):186–92.

14. Glazier RH, Klein-Geltink J, Kopp A, Sibley LM. Capitation and enhanced fee-for-service models for primary care reform: a population-based evaluation. Can Med Assoc J. 2009 May 26;180(11):E72–81.

15. Campbell DJT, Ronksley PE, Hemmelgarn BR, Zhang J, Barnabe C, Tonelli M, et al. Association of enrolment in primary care networks with diabetes care and outcomes among First Nations and low-income Albertans. Open Med. 2012 Dec 11;6(4):e155–65.

16. Lofters AK, Mark A, Taljaard M, Green ME, Glazier RH, Dahrouge S. Cancer screening inequities in a time of primary care reform: a population-based longitudinal study in Ontario, Canada. BMC Fam Pract. 2018 Dec;19(1):147.

17. Ouimet MJ, Pineault R, Prud’homme A, Provost S, Fournier M, Levesque JF. The impact of primary healthcare reform on equity of utilization of services in the province of Quebec: a 2003–2010 follow-up. Int J Equity Health. 2015 Dec;14(1):139.

18. Wei X, Li H, Yang N, Wong SYS, Owolabi O, Xu J, et al. Comparing Quality of Public Primary Care between Hong Kong and Shanghai Using Validated Patient Assessment Tools. PLOS ONE. 2015 Mar 31;10(3):e0121269.

19. Garcia-Subirats I, Vargas I, Mogollón-Pérez AS, De Paepe P, da Silva MRF, Unger JP, et al. Inequities in access to health care in different health systems: a study in municipalities of central Colombia and north-eastern Brazil. Int J Equity Health. 2014 Dec;13(1):1–15.

20. Thomson M. Who had access to doctors before and after new universal capitated subsidies in New Zealand? Health Policy. 2019 Aug;123(8):756–64.

21. Burström B, Burström K, Nilsson G, Tomson G, Whitehead M, Winblad U. Equity aspects of the Primary Health Care Choice Reform in Sweden - a scoping review. Int J Equity Health. 2017 Jan 28;16(1):29.

22. Beckman A, Anell A. Changes in health care utilisation following a reform involving choice and privatisation in Swedish primary care: a five-year follow-up of GP-visits. BMC Health Serv Res. 2013 Oct 31;13:452.

23. Kullberg L, Blomqvist P, Winblad U. Market-orienting reforms in rural health care in Sweden: how can equity in access be preserved? Int J Equity Health. 2018 Aug 17;17(1):123.

24. Kroll LE, Lampert T. Direct costs of inequalities in health care utilization in Germany 1994 to 2009: a top-down projection. BMC Health Serv Res. 2013 Dec;13(1):271.

25. Tiagi R. Access to and utilization of health care services among Canada’s immigrants. Int J Migr Health Soc Care Hove. 2016;12(2):146–56.

26. Chen LM, Farwell WR, Jha AK. Primary Care Visit Duration and Quality: Does Good Care Take Longer? Arch Intern Med. 2009 Nov 9;169(20):1866–72.

27. Vikum E, Bjorngaard JH, Westin S, Krokstad S. Socio-economic inequalities in Norwegian health care utilization over 3 decades: the HUNT Study. Eur J Public Health. 2013 Dec 1;23(6):1003–10.

28. Peckham A, Ho J, Marchildon G. Policy innovations in primary care across Canada. Rapid Rev. 2018;(1):63.

29. Sanmartin C, Berthelot JM, Ng E, Murphy K, Blackwell DL, Gentleman JF, et al. Comparing Health And Health Care Use In Canada And The United States. Health Aff (Millwood). 2006 Jul;25(4):1133–42.

30. Rivera LA, Henschke MT, Khoo E, Ing S, Bae SJ, Rice CM, et al. A modeling study exploring the impact of homelessness on rostered primary care utilization in Calgary, Canada. Can J Public Health. 2018 Aug;109(4):451–8.

31. Glazier RH. Balancing equity issues in health systems: perspectives of primary healthcare. Heal Pap. 2007;8 Spec No:35–45.

32. Victora CG, Joseph G, Silva ICM, Maia FS, Vaughan JP, Barros FC, et al. The Inverse Equity Hypothesis: Analyses of Institutional Deliveries in 286 National Surveys. Am J Public Health. 2018 Apr;108(4):464–71.

33. Loignon C, Hudon C, Goulet É, Boyer S, De Laat M, Fournier N, et al. Perceived barriers to healthcare for persons living in poverty in Quebec, Canada: the EQUIhealThY project. Int J Equity Health. 2015 Jan 17;14(1):4.

34. Bloch G, Rozmovits L, Giambrone B. Barriers to primary care responsiveness to poverty as a risk factor for health. BMC Fam Pract. 2011 Jun 29;12(1):62.

35. Wellstood K, Wilson K, Eyles J. ‘Reasonable access’ to primary care: assessing the role of individual and system characteristics. Health Place. 2006 Jun 1;12(2):121–30.

36. Agerholm J, Bruce D, Ponce de Leon A, Burström B. Socioeconomic differences in healthcare utilization, with and without adjustment for need: An example from Stockholm, Sweden. Scand J Public Health. 2013 May 1;41(3):318–25.

37. Asada Y, Kephart G. Equity in health services use and intensity of use in Canada. BMC Health Serv Res. 2007 Dec;7(1):41.

38. Cheng TC, Guo Y. Adult Immigrants’ Utilization of Physician Visits, Dentist Visits, and Prescription Medication. J Racial Ethn Health Disparities. 2019 Jun;6(3):497–504.

39. De Luca G, Ponzo M, Andrés AR. Health care utilization by immigrants in Italy. Int J Health Care Finance Econ. 2013 Mar;13(1):1–31.

40. Hamada S, Takahashi H, Sakata N, Jeon B, Mori T, Iijima K, et al. Household Income Relationship With Health Services Utilization and Healthcare Expenditures in People Aged 75 Years or Older in Japan: A Population-Based Study Using Medical and Long-term Care Insurance Claims Data. J Epidemiol. 2019 Oct 5;29(10):377–83.

41. Katz SJ, Hofer TP, Manning WG. Physician Use in Ontario and the United States: The Impact of Socioeconomic Status and Health Status. Am J Public Health. 1996;86(4):5.

42. Wang BR, Kwon YD, Jeon W, Noh JW. Factors associated with the frequency of physician visits among North Korean defectors residing in South Korea: a cross-sectional study. BMC Health Serv Res. 2015 Dec;15(1):90.

43. Turpel-Lafond ME. In Plain Sight: Addressing Indigenous-specific Racism and Discrimination in B.C. Health Care [Internet]. 2020 Nov [cited 2021 Sep 16]. Available from: https://engage.gov.bc.ca/app/uploads/sites/613/2020/11/In-Plain-Sight-Full-Report.pdf

44. Dryden O, Nnorom O. Time to dismantle systemic anti-Black racism in medicine in Canada. CMAJ. 2021 Jan 11;193(2):E55–7.

45. Peters G. Submission to the B.C. Government on Accessibility Legislation [Internet]. Broadbent Institute; 2019 Nov. Available from: https://d3n8a8pro7vhmx.cloudfront.net/broadbent/pages/57/attachments/original/1575399768/Submission_to_BC_Government_on_Accessibility_Legislation.pdf?1575399768

46. Chouinard V, Crooks * VA. ‘Because they have all the power and I have none’: state restructuring of income and employment supports and disabled women’s lives in Ontario, Canada. Disabil Soc. 2005 Jan;20(1):19–32.

47. Ministry of Health. Are you Eligible? [Internet]. Province of British Columbia; n.d. [cited 2021 Sep 21]. Available from: https://www2.gov.bc.ca/gov/content/health/health-drug-coverage/msp/bc-residents/eligibility-and-enrolment/are-you-eligible

48. British Columbia Ministry of Health [creator](2019): Consolidation File (MSP Registration & Premium Billing). V2. Population Data BC [publisher]. Data Extract. MOH(2019). http://www.popdata.bc.ca/data

49. British Columbia Ministry of Health [creator](2018): Medical Services Plan (MSP) Payment Information File. V2. Population Data BC [publisher]. Data Extract. MOH(2019). http://www.popdata.bc.ca/data

50. Canadian Institute for Health Information(2019): National Ambulatory Care Reporting System. V2. Population Data BC [publisher]. Data Extract. MOH(2019). http://www.popdata.bc.ca/data

51. British Columbia Ministry of Health [creator](2019): PharmaNet. V2. Population Data BC [publisher]. Data Extract. Data Stewardship Committee(2019). http://www.popdata.bc.ca/data

52. Canadian Institute for Health Information [creator](2019): Discharge Abstract Database (Hospital Separations). V2. Population Data BC [publisher]. Data Extract. MOH(2019). http://www.popdata.bc.ca/data

53. Peterson S, Wickham M, Lavergne R, Beaumier J, Ahuja M, Mooney D, et al. Methods to comprehensively identify emergency department visits using administrative data in British Columbia [Internet]. Vancouver, BC: Centre for Health Services and Policy Research; 2021 Feb. Available from: http://chspr.sites.olt.ubc.ca/files/2021/02/CHSPR-ED-Report-2021.pdf

54. Wilkins R. Use of postal codes and addresses in the analysis of health data. 1993 Jan 1;157–77.

55. Wilkins R. PCCF+ version 5E user’s guide: Automated geographic coding based on the statistics canada postal code conversion files, including postal codes through March 2009. [Internet]. Statistics Canada; 2009 Jul. Report No.: 82F0086-XDB. Available from: https://publications.gc.ca/collections/collection_2018/statcan/CS82-0086-2009-1-eng.pdf

56. Statistics Canada. Statistical Area Classification (SAC) [Internet]. 2018 [cited 2021 Sep 21]. Available from: http://www150.statcan.gc.ca/n1/pub/92-195-x/2011001/other-autre/sac-css/sac-css-eng.htm

57. Quan H, Li B, Couris CM, Fushimi K, Graham P, Hider P, et al. Updating and Validating the Charlson Comorbidity Index and Score for Risk Adjustment in Hospital Discharge Abstracts Using Data From 6 Countries. Am J Epidemiol. 2011 Mar 15;173(6):676–82.

58. British Columbia Ministry of Health, College of Physicians and Surgeons of BC. Medical Services Plan Practitioner File [Internet]. Population Data BC [publisher]; 2018 2019. Report No.: V2. Available from: http://www.popdata.bc.ca/data

59. Public Health Agency of Canada. Social determinants of health and health inequalities [Internet]. 2020 [cited 2021 Sep 21]. Available from: https://www.canada.ca/en/public-health/services/health-promotion/population-health/what-determines-health.html

60. Menec VH, Shooshtari S, Nowicki S, Fournier S. Does the Relationship Between Neighborhood Socioeconomic Status and Health Outcomes Persist Into Very Old Age? A Population-Based Study. J Aging Health. 2010 Feb;22(1):27–47.

61. Daskalopoulou SS, Khan NA, Quinn RR, Ruzicka M, McKay DW, Hackam DG, et al. The 2012 Canadian Hypertension Education Program Recommendations for the Management of Hypertension: Blood Pressure Measurement, Diagnosis, Assessment of Risk, and Therapy. Can J Cardiol. 2012 May;28(3):270–87.

62. Ivers NM, Jiang M, Alloo J, Singer A, Ngui D, Gall C. Diabetes Canada 2018 clinical practice guidelines. Can Fam Physician. 2019;65(1):11.

63. Canadian Institute for Health Information. How Canada Compares: Results From the Commonwealth Fund’s 2020 International Health Policy Survey of the General Population in 11 Countries. Ottawa, ON: CIHI; 2021.

64. Lee S, Mahl S, Rowe B. The Induced Productivity Decline Hypothesis: More Physicians, Higher Compensation and Fewer Services. Healthc Policy Polit Santé. 2021 Nov 26;17(2):90–104.

65. Lavoie JG, Varcoe C, Wathen CN, Ford-Gilboe M, Browne AJ, On behalf of the EQUIP Research Team. Sentinels of inequity: examining policy requirements for equity-oriented primary healthcare. BMC Health Serv Res. 2018 Sep 10;18(1):705.

66. Lavergne MR, King C, Peterson S, Simon L, Hudon C, Loignon C, et al. Patient characteristics associated with enrolment under voluntary programs implemented within fee-for-service systems in British Columbia and Quebec: a cross-sectional study. CMAJ Open. 2022 Jan;10(1):E64–73.

67. Morgan S, Evans RG, HanleY GE, Caetano PA, Black C. Income-Based Drug Coverage in British Columbia: Lessons for BC and the Rest of Canada. Healthc Policy. 2006 Nov;2(2):115–27.

68. El-Adam S, Ahuja M, Lavergne R, Peterson S, McGrail K. Alternative Payment Plan Remuneration Trends in British Columbia [Internet]. Vancouver, BC: Centre for Health Services and Policy Research; 2020 Sep. Available from: http://chspr.sites.olt.ubc.ca/files/2020/09/CHSPR-APP-Report-2020.pdf

69. British Columbia’s Office of the Human Rights Commissioner. Disaggregated demographic data collection in British Columbia: The grandmother perspective [Internet]. Vancouver, BC: BCOHRC; 2020 [cited 2021 Sep 21]. Available from: http://epe.lac-bac.gc.ca/100/200/300/bcohrc/disaggregated/BCOHRC_Sept2020_Disaggregated-Data-Report.pdf
